# A New Approach to Laminar Flowmeters

**DOI:** 10.3390/s101210560

**Published:** 2010-11-29

**Authors:** Fernando Lopez Pena, Alvaro Deibe Diaz, Marcos Rodriguez Lema, Santiago Vazquez Rodriguez

**Affiliations:** Integrated Group for Engineering Research, University of A Coruña, Escola Politécnica Superior, Mendizábal S/N, 15403 Ferrol, Spain; E-Mails: adeibe@udc.es (A.D.D.); marcos.lema@udc.es (M.R.L.); svr@udc.es (S.V.R.)

**Keywords:** flowmeter, laminar flow element, Poiseuille flow, entry length

## Abstract

After studying the performance and characteristics of actual laminar flowmeters a new disposition for this type of sensors is proposed in such a way that the measurement errors introduced by the intrinsic nature of the device can be minimized. The preliminary study shows that the developing entry region introduces non-linearity effects in all these devices. These effects bring about not only errors, but also a change in the slope of the linear calibration respect of the Poiseuille relation. After a subsequent analysis on how these non-linearity errors can be reduced, a new disposition of this type of flowmeters is introduced. This device makes used of flow elements having pressure taps at three locations along its length and connected to three isolated chambers. In this way, the static pressure can be measured at three locations and contributed to by the pressure taps at the level of each chamber. Thus the linearization error is reduced with an additional advantage of producing a reduced pressure drop.

## 1. Introduction

Flowmeters are devices of widespread use in many industrial processes that can use many different flows under many different conditions of pressure and temperature and can have many different requirements concerning cost, accuracy, safety, pressure losses, or materials compatibility, among others. A wide range of different types of flowmeters has been developed to satisfy the requirements in all cases regardless of these huge variations in fluid properties and circumstances [1]. The increasing request for better accuracy and easier automation has impelled the development of new types of flowmeters based *i.e.*, on Coriolis forces or ultrasound, as well as the improvement of classical ones, mostly by adding some electronics [2].

Merging electronics into classical types of flowmeters has been quite common in the last decades as a means of increasing sensor accuracy, easing their use and/or facilitating their inclusion in monitoring or control systems. This trend started by just replacing mechanical or pneumatic based secondary devices by transducers allowing the translation of the physical quantity being measured into an analog or digital signal ready to be acquired by an electronic processor or a computer. In some cases this trend evolved lately towards the inclusion of some modifications in the original sensor design in order to obtain further advantages out of the electromechanical merger. This is the case of the work presented here, where it is shown that introducing some modifications on the standard design of a laminar flowmeter can lead to the enhancement of its characteristics after adding a simple auxiliary electronic board.

Laminar flowmeters are a well-known kind of differential pressure-based flow measurement devices mainly used for measuring low flow rates of gases and liquids [3]. A laminar flowmeter consists in a laminar flow element and a differential pressure gauge or transducer. The laminar flow element guarantees that the flow passing through the flowmeter is in a laminar condition, that is, the flow exhibits no turbulence. In this condition viscous forces—generated by internal fluid friction—dominate over inertial forces and consequently the dominant mechanism for resistance to fluid motion is friction against the surrounding walls. The parameter characterizing this type of flows is the Reynolds number, defined as the ratio between inertial and viscous forces. For a fluid of density *ρ* and viscosity *μ* moving along a pipe of diameter *D* and having a volumetric flow rate *Q*, the Reynolds number is expressed as:

(1) $$\text{Re}=\frac{\rho QD}{\mu A}=\frac{4\rho Q}{\mu \pi D}$$

where *A* = *πD*^2^/4 is the pipe cross-sectional area. Under normal operating conditions and for values of the Reynolds number below 2,300, the flow remains laminar. The pressure drop (Δ*p*) created by fluid friction between two points separated a distance Δ*x* along a pipe in a laminar flow regime is quantifiable, and can be expressed by the Hagen-Poiseuille equation:

(2) $$\Delta P=\frac{128\mu Q\Delta x}{{\pi D}^{4}}$$

This equation establishes that for a laminar flow there is a linear relationship between flow rate and developed pressure drop; this linearity represents an advantageous characteristic of laminar flowmeters. A major drawback of this type of flowmeter, however, is its dependence on fluid viscosity, which in turn is mostly dependent on fluid temperature. Thus, any laminar flowmeter requires some form of temperature compensation to obtain precise measurements.

**Figure 1.**
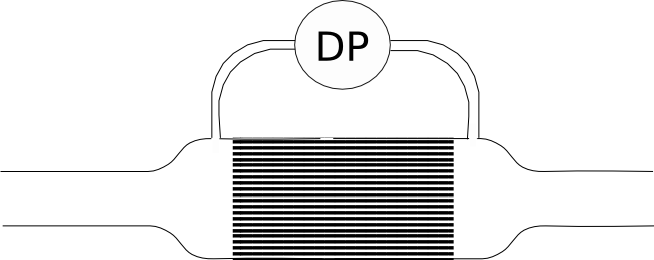
A typical laminar flowmeter.

A laminar flow element can be constructed by various methods, but most commonly it consist of a set of capillary ducts whose length significantly exceed their inner diameter, and that are arranged in parallel. In this way the main flow is split among all of them obtaining, as a result, a reduced Reynolds number. To help in this reduction, very often the sum of the cross-sectional area of all capillaries is larger than the main pipe cross-sectional area. Figure 1 represents a flowmeter having one of these laminar flow elements.

There are three additional sources of pressure drop in this type of flowmeter that introduce nonlinearity and error to the capillary loss. These are: Inlet loss, exit loss, and capillary entrance loss. The inlet loss is produced by the effects of flow velocity changes when entering the pipe, as well as inlet edge effects on the flow [4]. At the capillary exit the abrupt change in the effective cross-sectional area of the flow emerging from the capillaries produces a momentum loss [5]. The entrance loss refers to the losses resulting from the transformation of the flow velocity profile from uniform at the inlet to the characteristic laminar flow profile at a certain distance called entrance length [6]. Inlet and exit losses can be greatly reduced by a careful design, but entrance loss is intrinsic of this kind of pipe flows.

In order to reduce the effects of this non-linear source of error, the Reynolds number inside the capillaries is commonly kept below 1,200 and the *L*/(Re*D*) ratio above 3 [3]. In any case, and as pointed out by Siev *et al.* [3], to measure the true capillary differential pressure drop according to the Poiseuille equation, it would be necessary to insert the pressure taps in the capillary at the calculated L dimension. They considered that this is impractical because of the small tubing used in this type of flowmeters. However, this is the approach explored in the present work as will be explained later. The idea is to use relatively large pipe diameters (of around 3 mm), increase the Reynolds number to the maximum allowable of 2,000 to compensate for the reduction in pressure drop, and providing some means to overcome the difficulties related with the consequent increment of entry length. In the following section we are going to highlight the main concepts related with entry effects that support our proposal prior to detailing the proposed approach in the subsequent sections.

## 2. Considerations on Entry Length Effects

It is customary to perform this analysis using non-dimensional variables. All lengths are normalized by the diameter *D*. Thus the non-dimensional length along the pipe is *x*’ = *x*/*D*, but in some cases the form *X* = *x*/(Re*D*) is used for convenience. The pressure is normalized by *ρU*^2^/2, obtaining a dimensionless pressure in the form: *p*’ = *p*/(*ρU*^2^/2). Using these dimensionless variables, from expressions 1 and 2 the following expression valid for fully developed Poiseuille flow is obtained:

(3) $$\frac{dp^{\prime }}{dX}=-64$$

A measure of the total pressure drop from the pipe inlet will include a term accounting for the fully developed flow plus the excess pressure drop *K* accounting for the entry region:

(4) $$\Delta p^{\prime }=64X+K(X)$$

**Figure 2.**
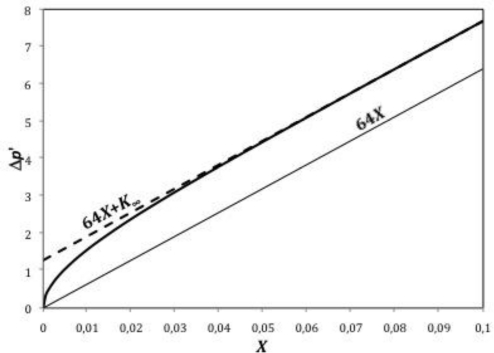
Pressure drop development along a pipe.

The term *K*(*X*) rises asymptotically along the entry region from zero at *x* = 0 to a constant value *K*_∞_ in the developed region. Figure 2 represents Δ*p*’(*X*) for a real case with Re = 500 and compares it with a hypothetical case following a Poiseuille flow without entry effects. Its asymptotical trend towards a fully developed flow can be clearly appreciated.

According to White [7], it can be considered that *K* approaches its final value *K*∞ when *X* ≈ 0.08. Ideally, and in order to obtain perfectly linear measurements, the pressure drop should be measured between pressure taps located at *X* > 0.08. Taking into account that we are seeking to use pipes of few millimetres in diameter this limitation would lead to laminar flow elements being very long.

There are two main ways to reduce this length; the first one is by reducing the pipe diameter and the second one by allowing a small level of non-linearity in the device response. The limitations to the first approach are related to the need of drilling holes on the pipe walls, while in the second case the required accuracy will be the constraint.

Shah [8] makes use of data from previous works of several researchers to provide empirical correlations for the apparent friction coefficient in the entry length of circular and non-circular ducts. Particularizing his formula for circular pipes, an expression relating K with X can be easily derived as:

(5) $$K=X\frac{1.25X+\frac{0.002917}{\sqrt{X}}-0.013568}{X^{2}+0.000212}$$

**Figure 3.**
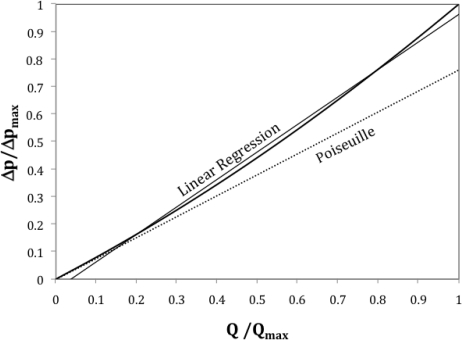
Flowmeter response and two possible linearization paths.

Any laminar flowmeter, as in Figure 1, can be characterized by using expressions 1, 4 and 5, in addition to the restriction of the maximum allowed value for the Reynolds number. A non-dimensional form of the total pipe length *L* as *L*/(Re*D*) is the best parameter indicating the linearity of the device, as stated in [3]. Figure 3 presents the response of a flowmeter with *L*/(Re*D*) = 0.06, together with its ideal linear response for a Poiseuille flow without entrance effects and the linear regression of its actual response curve, which is used here as Best Straight Line (BSL) to fit.

**Figure 4.**
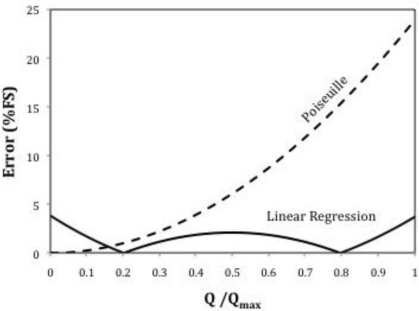
Linearization errors corresponding to Figure 3.

Figure 4 represents the errors achieved when these linear approximations are used as response. Obviously the linear regression performs much better than the Poiseuille approximation except at very low flow rates. In order to reduce these errors, Siev [3] recommends the use of a minimum value of 0.3 for *L*/(Re*D*), and preferably of 0.6 or greater, so that the entry length errors become negligible.

**Figure 5.**
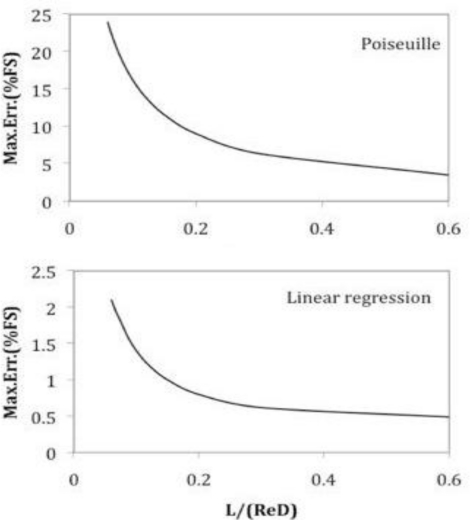
Nonlinearity errors achieved by the linear approximations.

Figure 5 represents the maximum nonlinearity error achieved with both linear approximations as a function of *L*/(Re*D*). The decreasing trend is quite similar in both cases, but the error obtained when the linear regression is used consistently appears to be roughly ten times smaller than that of the Poiseuille approximation.

**V** It is generally advised that a calibration of the flowmeter should be performed instead of using the Poiseulle relation (2) directly, due to the fact that in this expression the diameter *D* of the pipes appears to the fourth power, thus any uncertainty in determining this value will result in a significant error. Being this statement true, the analysis presented above shows that even when the true value of the diameter *D* is known, the use of the Poiseuille relation would introduce significant errors, while performing a linear regression of the calibration data points would reduce the error by one order of magnitude.

**Figure 6.**
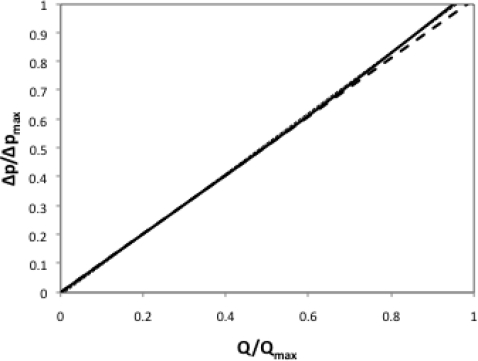
Flowmeter response after introducing a pressure tap (solid line) and its linear Poiseuille model (dashed line).

In the case of measuring the pressure drop between two pressure taps on the pipe wall; one at position *x*_1_ downstream the entrance and the other at the pipe end, the response curve of Figure 3 should change as in Figure 6.

**Figure 7.**
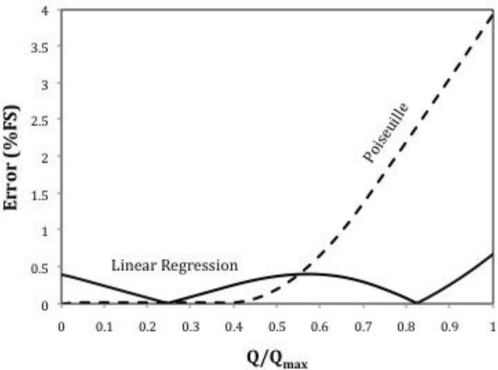
Linearization errors corresponding to Figure 6.

In the present case the coordinate *x*_1_ has been placed such that *x*_1_/*L* = 0.14. The corresponding linearization errors are shown in Figure 7. Notice that the Poiseuille model produces negligible errors until the entrance length reaches position *x*_1_, in addition its maximum value is now one sixth of the original one. On the other hand, the linear regression error has decreased by one order of magnitude.

One way of avoiding these non-linearity effects could be achieved by placing the first pressure tap at a distance larger than the entrance length, corresponding to the maximum Reynolds number allowed. As we can see now, this approach will result in very long devices. In fact, using the limit value of Re = 2,000, and as according to White [7] the entry length *x*_L_ defined as the point where *K* approaches its final value, occurs at *x*_L_/(Re*D*) = 0.08, which implies that the pressure tap should be placed at least at *x*_L_/*D* = 160. Taking into account that in order to produce adequate pressure taps the pipe diameter should be several millimetres, and taking for instance 3 mm of diameter, this gives a distance of 480 mm from the pipe inlet. The total pipe length must be such as to allow a free distance downstream of this position, so that a pressure drop equal to the full scale of the pressure transducer used may be obtained. Even in the case of using a low-pressure transducer, the total length of the device would easily exceed 700 mm, which can be considered excessive for many applications.

## 3. Proposed Flowmeter Disposition

As shown in the previous section, entry effects are always present in the performance of any laminar flowmeter having the classical disposition shown in Figure 1. These effects manifest themselves in two ways; firstly they modify the coefficient of the Poiseuille linear relation, and secondly they introduce a non-linearity error. The reduction of these unwanted effects obtained by increasing the *L/*(Re*D*) ratio is larger for the non-linearity effect. In a traditional design this value is increased up to a point where these non-linearity effects are negligible. Another interesting tip is that by placing a pressure tap at a deep position inside the pipe it will be possible to obtain a perfectly linear Poiseuille flowmeter, but this will result in a very long device.

Taking these considerations into account, the modifications on the flowmeter design proposed here consider the non-linearity errors and try to reduce them to a required level. This will be achieved by placing several pressure taps along the flow element pipes, which require a pipe diameter of few millimetres. The *L*/(Re*D*) value is going to be one tenth of the one used in a traditional design, trying to reduce *L*/*D* while preserving the Reynolds number close to the maximum that leads to a laminar flow. In this way the device could have a reasonable length while the number of pipes needed could be reduced. Another advantage of this philosophy is the potential reduction of the total pressure drop along the device.

**Figure 8.**
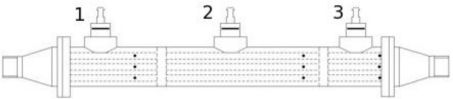
Proposed flowmeter arrangement.

Following the proposed approach, and in order to produce correct measurements, three pressure taps are placed along one or several of the laminar flow element tubes. The tubes are arranged as if they are a part of a “shell and tube” heat exchanger but having two extra intermediate walls, resulting in three chambers along the shell, as sketched in Figure 8. Each one of them will provide the pressure contributed by the part of the tubes having one of their pressure taps at the level of this chamber. Thus the chamber will also equalize the pressure of the various taps it contains thus diminishing the effects of partial blockage of some of them. The potential total blockage of some of the taps will produce no effects on the measurement, as the flowmeter can perfectly work when having just one working tap per chamber. As, on the other hand, the pipes have a relatively large diameter, it doesn’t appear at first that this apparatus will need more periodical cleaning than a regular laminar flowmeter.

The idea is to use the pressure difference between chambers 1 and 3 to measure low flow rates and switch to 2 and 3 at higher flow rates, at a point where entrance effects begin producing significant non-linearities at position 1. The distance from the pressure taps in chamber 3 to the pipe end can be just of about two pipe diameters, as this distance is enough to avoid exit losses. The distance between pressure taps in chambers 2 and 3 must be such as to produce a pressure drop equal to the full span of the pressure transducer used when the maximum allowed Reynolds number is achieved. The distance between the inlet and the pressure taps at position 2 must be such that at the maximum allowed value for the Reynolds number, the entry effects at this position result in an error level below the value imposed in the design. The pressure taps in chamber 1 should be placed in a position such that when the pressure drop between 1 and 3 reaches the full range of the transducer, the entry effects at position 1 introduce an error below the maximum allowed.

**Figure 9.**
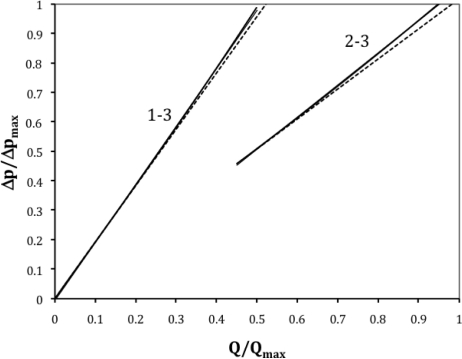
Response of the proposed flowmeter.

Figure 9 represents the response of the proposed flowmeter. At low flow rates the differential pressure is measured between 1 and 3. When the pressure transducer produces values close to its full scale, the measurement is switched to 2–3 until the maximum flow rate is reached.

**Figure 10.**
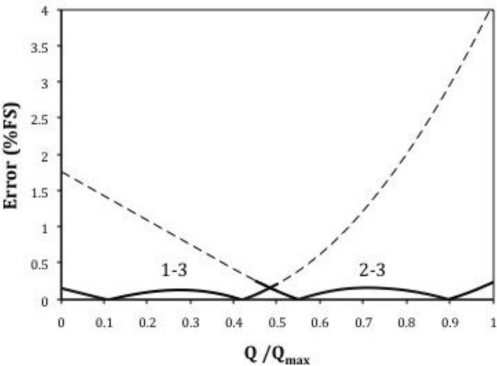
Linearization errors of the proposed flowmeter.

Figure 10 represents the linearization errors achieved with this arrangement. The maximum non-linearity error is of 0.12%. Obviously the Poiseuille relation is not used here for the reasons explained above.

Another advantage of the proposed setup is that the full range of the pressure transducers is used to measure half of the range of the flow rate, thus the errors referred to the transducer full scale are now divided by two when referring to the flowmeter’s full scale. Nevertheless, the only way of making a functional instrument based on this concept will be to introduce an electronic unit able to execute adequate switching between both halves of the full range and give a correct single output. This aspect of the device is clarified in the next section.

Notice that in the example presented above, the maximum allowed value for the Reynolds number has been chosen. Reducing this value or reducing the diameter of the flow element pipes will produce a proportional reduction on the maximum non-linear error, in the same way that they do for conventional laminar flowmeters. Both reductions will have an effect of decreasing the maximum flow rate per pipe, thus the number of pipes needed for a given total flow rate should be increased.

## 4. Electronic Unit

**Figure 11.**
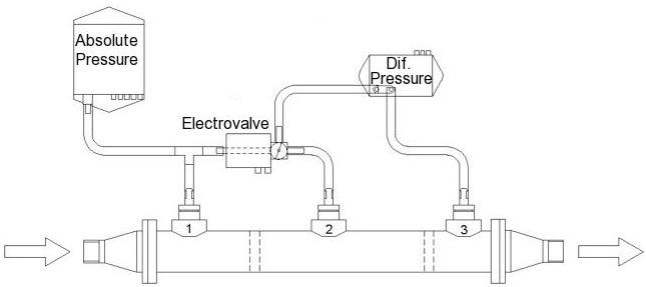
Pressure transducers and pneumatic connections disposition.

Prior to defining the electronic unit and its functions, it is necessary to decide the disposition of the pressure transducers. One possibility is to permanently connect two pressure transducers; one between pressure connectors 1 and 3 and the second one between 2 and 3. In this case the first pressure transducer should be able to support the overpressure suffered at the high flow rate range, where the other transducer is performing the task. Another possibility is to use a single pressure transducer having its low-pressure port directly connected to pressure connector 3 and an additional electrovalve in charge of switching connectors 1 or 2 to the high-pressure port. Figure 11 represents this setup. This figure depicts the total or gauge pressure transducer needed to determine the flow density when measuring gas rates. As in regular commercial laminar flowmeters, a temperature sensor is also needed to determine gas density and, in some cases, a relative humidity sensor is added to correct this value [9].

**Figure 12.**
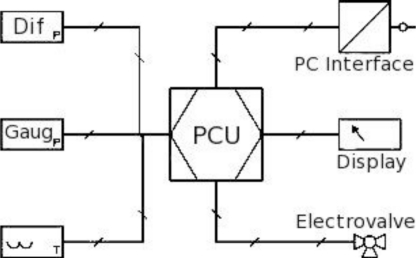
The flowmeter’s electrical block diagram.

The electric diagram for the device is presented in Figure 12. A Process and Control unit (PCU) takes signals coming from the differential and the total or gauge transducers, from the temperature sensor and, optionally, from a humidity sensor. The PCU, after processing the signals, controls the electrovalve, changing its state if needed, and finally it presents the actual flow measurement in a display and sends the information in digital form to a processing system or PC.

The PCU can have some linearization programs implemented. In this case the programs should take into account the non-linearities introduced by the differential pressure transducer in addition to those of the LFE. However, maintaining the LFE non-linearity error below a given value will be enough in most applications. One interesting point here is that the response curve of practically any pressure transducer has a negative second derivative, that is, its slope diminishes as the pressure goes higher, which is the opposite of the LFE. Consequently the combined non linearity errors will try to compensate each other and taking the higher of both values as the non-linearity error of the assembly would be a conservative approach.

## 5. Conclusions

After studying the performance of actual laminar flowmeters, it is found that the developing entry region introduces non-linearity effects even in cases when the *L*/(Re*D*) parameter is high. As a consequence, non-linearity errors and change in the slope of the linear calibration appear.

A new arrangement of this type of flowmeters, with flow elements that include pressure taps along their pipes, has been introduced. The proposed setup splits the full flow rate range into two parts, reducing in this way the linearization error, with the additional advantage of producing a reduced pressure drop and a better use of the pressure sensor range.

This proposal accounts for the non-linearity error of the device and permits limiting its level at design, in order to pair it with the accuracy of the flowmeter and the rest of its components. A fully linear device is also proposed at the cost of increasing its length.

A general description of the device, as well as a discussion on the setup, its associated transducers, pneumatic connections and electric scheme is presented. The fields of application of the proposed flowmeter remain the same as for conventional laminar flowmeters, *i.e*., those typically related to low rate gas flow measurements.
